# Alterations promoted by acid straightening and/or bleaching in hair microstructures

**DOI:** 10.1107/S1600576723005599

**Published:** 2023-08-01

**Authors:** C. R. R. C. Lima, R. J. S. Lima, A. C. C. Bandeira, R. A. A. Couto, M. V. R. Velasco, H. N. Bordallo, C. L. P. Oliveira

**Affiliations:** a Institute of Physics, University of São Paulo, São Paulo 05508-090, SP, Brazil; b Academic Unit of Physics, Federal University of Campina Grande, Campina Grande 58429-900, PB, Brazil; c Niels Bohr Institute, University of Copenhagen, 2300 Copenhagen, Denmark; d Institute of Chemistry, University of São Paulo, São Paulo, Brazil; e Faculty of Pharmaceutical Sciences, University of São Paulo, São Paulo 05508-000, SP, Brazil; f European Spallation Source ESS ERIC, PO Box 176, SE-221 00 Lund, Sweden; University of Sydney, Australia

**Keywords:** hair, acid straightening, bleaching, damage, X-ray scattering, microstructure, cosmetic treatments, neutron spectroscopy, biopolymers

## Abstract

Human hair is a biopolymer constituted mainly of keratin intermediate filaments, lipids, pigments and water. Cosmetic treatments usually interact with the hair at the molecular level, inducing changes in its components and modifying the physicochemical and mechanical properties of the fibers. Here, the effect of acid straightening on the morphology and ultrastructure of Caucasian hair was investigated by a group of complementary experimental methods: wide-, small- and ultra-small-angle X-ray scattering; high-resolution 3D X-ray microscopy; quasi-elastic neutron scattering and inelastic neutron scattering; thermogravimetry–mass spectrometry; and differential scanning calorimetry (DSC). X-ray diffraction patterns showed that acid straightening associated with a flat iron (∼180°C) changed the cortex of the fiber, shown by denaturation of the intermediate filaments (measured by DSC). The increase in the spacing of the lipid layers and the observation of the dehydration behavior of the fiber provided indications that water may be confined between these layers, while neutron spectroscopy showed alterations in the vibration mode of the CH_2_ groups of the lipids and an increase of the proton (H^+^) mobility in the hair structure. The latter may be associated with the extremely low pH of the formulation (pH ≃ 1). Additionally, this investigation showed that bleached hair (one-time bleached) is more damaged by the action of acid straightening than virgin hair, which was shown by a threefold increase in the percentage of total porosity of the tresses. The obtained results demonstrate that the investigation approach proposed here can provide very important thermodynamic and structural information on induced changes of hair structure, and certainly can be applied for the evaluation of the action mode and efficiency of cosmetic treatments.

## Abstract 2

A systematic investigation on alterations promoted by acid straightening and/or bleaching in hair microstructures is presented. By combining X-ray scattering methods and microcomputed tomography, among several other methods, innovative results were obtained on the structure and thermodynamics of human hair subjected to cosmetic procedures.

## 1. Introduction

Feughelman (1959 ▸) proposed a model for human hair in which the α-helix aggregates in intermediate filaments (IFs), forming a crystalline continuous axially oriented phase, are embedded in an amorphous matrix that comprises the proteins associated with IFs. A hair fiber is mainly composed of three distinct regions: cortex, cuticle and cell-membrane complex (CMC) (Robbins, 2012 ▸). A set of IFs form a very complex structure called a microfibril (Parry, 1995 ▸), localized in the cortex, promoting strength, resistance and elasticity of hair (Robbins, 2012 ▸). A united set of microfibrils form a macrofibril.

The cuticle (external barrier) contains 6–10 overlapping layers. Its function is to protect the cortex from environmental damage, and it promotes the hair brightness. The cuticle layers and cortex structures are held together by the CMC, which is formed by lipids and proteins. The lipids comprise cholesterol, cholesterol esters, cholesterol sulfate, free fatty acids, triglycerides, paraffin, squalene and ceramides (internal lipids) (Bouillon & Wilkinson, 2005 ▸). Covalently attached to the cuticle surface (external), there is the lipid 18-MEA, which protects the hair against damage from chemical treatments like bleaching (Masukawa *et al.*, 2005 ▸; Kon *et al.*, 1998 ▸). Changes in these structures modify the penetration of cosmetic products into the hair matrix as well as influencing the water content (Murthy *et al.*, 2019 ▸) and the mechanical properties (Robbins, 2012 ▸).

The demand for changes in the color and shape of hair has been increasingly frequent in haircare treatments, even with the occurrence of damage to the hair fibers caused by one or several concurrent cosmetic procedures, such as bleaching, straightening and thermal treatments. Chemical and physical damage on the hair fibers caused by using these treatments has already been described (Wolfram *et al.*, 1970 ▸; Baias *et al.*, 2009 ▸; Lima *et al.*, 2019 ▸; Wortmann *et al.*, 2002 ▸; Colenci, 2017 ▸; Martins Junior, 2015 ▸; Kon *et al.*, 1998 ▸; dos Santos *et al.*, 2019 ▸). Hair bleaching degrades melanin which causes irreversible physicochemical changes, producing cysteic acid residues by oxidation of the cystine amino acids (Wolfram *et al.*, 1970 ▸; Robbins, 2012 ▸). Structural studies using diffraction techniques applying wide- and small-angle X-rays (Stanić *et al.*, 2015 ▸; Song *et al.*, 2019 ▸) and neutrons (Murthy *et al.*, 2019 ▸; Kamath *et al.*, 2014 ▸; Lima *et al.*, 2020 ▸) have been used to investigate treated hair.

Traditional hair-straightening actives, already used worldwide for decades, are presented in alkaline formulations, and their mechanism of action is well known and disclosed in the literature (de Sá Dias *et al.*, 2007 ▸). In recent years, some compounds have been used in acid formulations to straighten hair, such as glyoxylic acid and its derivatives, including glyoxylic acid associated with carbocysteine and amino acids. Currently, these products are marketed in some countries, including Brazil, regardless of not being included on the ‘List of allowed actives for cosmetic products for straightening or waving the hair’, established by ANVISA (2023 ▸) via NI No. 220 of 13/04/2023 (RDC, Resolucão de Diretoria Colegiada, No. 409 of 27/07/2020), which discriminates the allowed actives: thio­glycolic acid and its salts, thio­glycolic acid esters, sodium or potassium hydroxide, lithium hydroxide, calcium hydroxide associated with guanidine salt, sulfites and inorganic bis­ulfites, and pyrogallol and thiol­actic acid.

The mechanism of action of these acid actives in hair straightening is still not well understood, and information showing the impact caused in the hair fiber by their use and the consequences of routine treatment by these substances is scarce. In general, the studies published to date suggest that reaction with an active straightener induces conformational rearrangements in the cortex and the cuticle (Taddei *et al.*, 2015 ▸; Boga *et al.*, 2014 ▸; Lima *et al.*, 2019 ▸).

Under a different perspective than those already described in the literature, we investigated the impact of acid straightening using the active *Glyoxyloyl Carbocysteine (and) Glyoxyloyl Keratin Amino Acids (and) Water* in the microstructures of hair fibers. Besides several results, we show the location of the damage in the hair structure promoted by acid straightening. The interaction of bleaching and this type of straightening is also evaluated.

## 2. Experimental details

### 2.1. Sample preparation

Caucasian untreated dark brown hair, obtained from a commercial source (DeMeo Brothers, New York), was formed into tresses (2 g and 10 cm long), washed (37.0 ± 5.0°C) with a 10%(*w*/*w*) dispersion of sodium ether lauryl sulfate and dried at room temperature for at least 48 h at 22.0 ± 2.0°C under 55% relative humidity. The hair tresses were divided into four groups: virgin/natural hair (VH), bleached hair (BH), straightened hair with formulation at pH 1.0 (SH), and bleached and straightened hair with formulation at pH 1.0 (BSH).

### 2.2. Straightener formulation preparation

The formulation was prepared as an oil-in-water emulsion, with the following composition according to International Nomenclature of Cosmetics Ingredients (INCI) name: *Aqua, Behentrimonium Metho­sulfate (and) Cete­aryl Alcohol, Iso­propyl Palmitate, PEG-90M, Polyquaternium-67, Shea Butter Amido­propyl Trimonium Chloride, Glyoxyloyl Carbocysteine and Glyoxyloyl Keratin Amino Acids* (15.0%; AQIA). The pH of the formulation was adjusted to 1.0 with citric acid.

### 2.3. Treatments

#### 2.3.1. Bleaching

The selected hair tresses were bleach damaged using a commercial treatment product based on an alkaline solution (pH 10.5) in the oxidant medium of hydrogen peroxide [20%(*v*/*v*)] and ammonium persulfate, applied for 30 min and at room temperature.

#### 2.3.2. Straightening

The tresses were treated with a ratio of 1.0 g of formulation/1.0 g of hair, according to their group classification, and the treatment was gently applied on the tresses with a brush so that it could be in contact with all the fibers for 20 min. Then, they were brushed, dried with a hairdryer and flattened ten times with a flat iron (180°C).

### 2.4. Methods

#### 2.4.1. X-ray scattering

Wide-, small- and ultra-small-angle X-ray scattering (WAXS, SAXS and USAXS, respectively) measurements were performed on an Xeuss 2.0 from Xenocs, with microfocus GeniX3D sources (Cu *K*α, λ = 1.54 Å; Mo *K*α, λ = 0.71 Å; and Cr *K*α, λ = 2.26 Å), FOX3D collimation optics and two sets of scatterless slits 2.0. Two-dimensional scattering intensities were collected on a PILATUS 300K detector with parameters according to Table 1 ▸. X-ray scattering data were collected in a transmission geometry and the hair fibers were all carefully positioned parallel along the axis of the hair. The apparatus used to mount the hair fibers in the experiment is shown in Fig. S1 of the supporting information. Since there was anisotropic scattering, due to the alignment of the hair tresses, it was necessary to perform sector analysis on the 2D images. The program package *FIT2D* (Hammersley, 2016 ▸) was used to perform azimuthal and radial integrations in order to produce one-dimensional curves of the scattering intensity as a function of the modulus of the momentum transfer, *q*, defined as 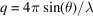 , where 2θ is the scattering angle. The program package *SUPERSAXS* (Oliveira *et al.*, 2009 ▸) was used for standard data-treatment procedures. The Xenocs Xeuss collects the forward beam that passes through the sample and the integration over this peak gives the normalization factor for the collected data. The ‘blank’ scattering for the background subtraction was the empty cell. The ranges of *q* for each sample–detector distance are shown in Table 1 ▸.

#### 2.4.2. Temperature-variation SAXS measurements

Small hair tresses (about 30 fibers) of the groups VH, BH and SH were joined and oriented vertically in a dedicated sample holder containing a device allowing heating of the tresses in a controlled manner. The sample holder was inserted in NANOSTAR equipment from Bruker, which has a microfocus source GeniX3D (Cu *K*α, λ = 1.54 Å), collimation optics FOX3D and two sets of scatterless slits 2.0, all provided by Xenocs. The sample–detector distance was 67 cm and the 2D data were obtained using a Bruker VÅNTEC-2000 detector. This detector is a 2D gaseous wire detector developed by Bruker with a resolution of ∼80 µm for each pixel size on a matrix of 1024 × 1024 pixels. Azimuthal and radial integrations on the 2D SAXS images were performed using the program provided in the Bruker system. The program package *SUPERSAXS* was also used for standard data-treatment procedures. In this case, the normalization factor for the experimental data is obtained by the use of a semitransparent beamstopper. The integral of the intensity collected behind the beamstopper provided the normalization factor (Oliveira *et al.*, 2009 ▸; Pedersen, 2004 ▸). The empty cell was used as a ‘blank’ for the background subtraction. The mounting of the hair tresses on the sample holder is shown in Fig. S2.

#### 2.4.3. Neutron spectroscopy

Hydrogen mobility in hair samples was investigated using quasi-elastic neutron scattering (QENS) and inelastic neutron scattering (INS) by means of the elastic fixed window (EFW) method (Lauritsen *et al.*, 2018 ▸). Using this methodology, it is possible to assess the evolution of neutrons that are elastically scattered by the sample as a function of temperature. Analysis of the elastic scattering response allows determination of the onset of proton mobility by the points of inflection in the collected data. The mobility of the hydrogen in the hair matrix was measured between 10 and 300 K with data collected upon heating. The data, collected at the backscattering spectrometer IRIS (Campbell *et al.*, 2000 ▸), located at the ISIS facility in the UK, provide an elastic energy resolution of 17.5 µeV at full width at half-maximum (FWHM). The data were collected for an upper experimental observation time of ∼200 ps and normalized to the response observed at the lowest temperature. Information on molecular vibrations occurring over a broad energy range from 1 to 1000 meV, corresponding to the femtosecond domain, was obtained using the indirect-geometry time-of-flight neutron spectrometer TOSCA (Pinna *et al.*, 2018 ▸), also located at ISIS. INS spectra for all samples were recorded at 10 K. During these experiments, the samples were confined in flat-plate aluminium containers sealed with indium wire. The collected data were converted to the incoherent dynamic structure factor, *S*(*Q*, ω), using the *Mantid* software (Arnold *et al.*, 2014 ▸). The data were normalized to sample mass and subsequently to the intensity of the elastic line.

#### 2.4.4. Thermogravimetry–mass spectrometry (TG–MS) analysis

The hair snippets were inserted into an open Al_2_O_3_ crucible containing 9.5–10.0 mg of sample. The TG–MS analysis was performed on an STA 409 PC Luxx (Netzsch) simultaneous TG/DSC instrument and a QMS 403C Aëolos (Netzsch) MSD mass spectrometer. Thermogravimetric curves and mass spectra were collected in duplicate using a heating rate of 10°C min^−1^ between 25 and 1000°C under dynamic air atmosphere (50 ml min^−1^).

#### 2.4.5. Differential scanning calorimetry (DSC)

DSC measurements, performed in triplicate, of hair samples immersed in water were conducted on a power-compensated instrument (DSC 6000, PerkinElmer, USA), using stainless steel large-volume pans, which are pressure resistant up to 25 bar. The temperature range was 50–180°C with a heating rate of 10°C min^−1^ (Wortmann *et al.*, 2002 ▸). Hair snippets (∼5 mg) were weighed into the DSC pans and 40 µl of water was added. The pans were sealed and stored overnight prior to the DSC measurements.

#### 2.4.6. High-resolution 3D X-ray microscopy (HRXRM) and data processing

Hair fibers (VH, BH, SH and BSH) were imaged using a 3D X-ray microscope (ZEISS Xradia Versa XRM-510) with an X-ray source voltage of 50 kV. In order to have the best sample magnification/framing ratio, the sample-to-detector and source–sample distances were 15 and 7 mm, respectively, with the reference position at the sample holder. This improved resolution using the 20X objective, placed just before the CCD camera, was necessary to measure the dimensions, in a total scan time of 3 h 30 min. The detector pixel size was 0.9 µm, which gives an overall spatial resolution of ∼3 µm. The exposure times per radiograph were 10 s. Approximately 1000 views were collected for each scan as the sample rotated over 360°, with a detector resolution of 1024 × 1024 pixels and a field of view of 900 µm. Volumetric data were reconstructed from the obtained scan data using the software *Avizo* 9 (Thermo Fisher Scientific) and *X Reconstructor* (ZEISS Xradia), with center shift and beam-hardening corrections.

## 3. Results and discussion

### 3.1. X-ray scattering

**Figure 1:**
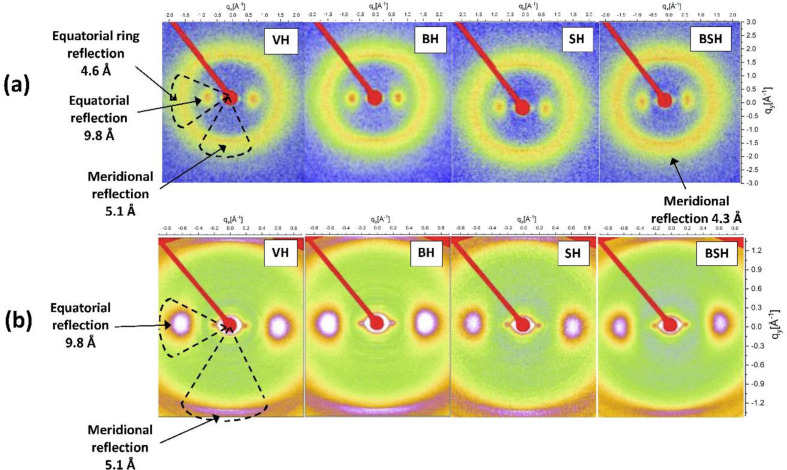
2D images obtained from WAXS zone planes on VH, BH, SH and BSH. The instrument setup: sample–detector distance = 14.3 cm, detector = (*a*) Mo (λ = 0.71 Å) and (*b*) Cu (λ = 1.54 Å).

WAXS and SAXS patterns were obtained in this work to evaluate alterations in the organized structure of human hair prior to and after cosmetic treatments. Figs. 1 ▸(*a*) and 1 ▸(*b*) shows the 2D images obtained from WAXS using Mo (λ = 0.71 Å) and Cu (λ = 1.54 Å) X-ray sources, respectively. Visual differences in the intensities are observed, indicating that the treatments affect the molecular organization within the hair. As seen from the 2D scattering images, there are regions with higher intensity (peaks), which are related to periodic distances inside the structure. For each sample, the integrations indicated in Fig. 1 ▸(*a*), along the equatorial (perpendicular to the fiber axis) and meridional (along the fiber axis) directions, were performed in order to analyze the specific regions already identified in the literature (Wade *et al.*, 2013 ▸; Coderch *et al.*, 2007 ▸).

**Figure 2:**
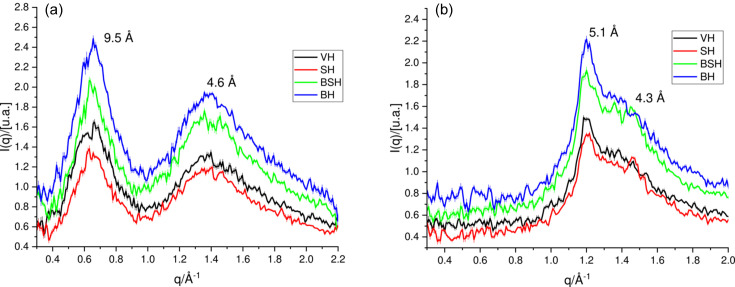
Integrations of the 2D scattering data in the WAXS zone planes with a sample–detector distance of 14.3 cm and Mo X-ray radiation: (*a*) equatorial and (*b*) meridional reflections for VH, BH, SH and BSH.

With this procedure, one obtains 1D curves of intensity as a function of *q*. These plots are shown in Figs. 2 ▸(*a*) and 2 ▸(*b*) (equatorial and meridional, respectively) with the profiles obtained from the 2D wide-angle scattering region (*q* range 0.01–2.0 Å^−1^), for all hair types. Two reflections (*d* spacings) are visible in the equatorial and meridional patterns in all types of hair: at 9.5 Å (along the axis of the fiber) and 5.1 Å (the axial repeat spacing). The features of these patterns correspond well to the known *d* spacing between adjacent coiled coils (Fraser *et al.*, 1964 ▸; Busson *et al.*, 1999 ▸; Kreplak *et al.*, 2004 ▸) or to the distance of chains from other structures (Kreplak *et al.*, 2004 ▸) and the superhelical structure of α-helices twisting around each other within coiled coils (regular α-helical coiled-coil folding), respectively (Crick, 1952 ▸; Cohen & Parry, 1994 ▸; Lupas & Gruber, 2005 ▸). Most importantly, the 5.1 Å meridian arc becomes less evident in straightened treated hair and another arc with a more intense meridional signal at 4.3 Å appears.

Cornwell *et al.* (1994 ▸) related the absence of a clear α-helix reflection at 5.1 Å to clear evidence in favor of the β form. Zhang *et al.* (2015 ▸) reported that a reduction in the α-helical signals at 9.6 and 5 Å (equatorial and meridional directions, respectively) in comparison with the lipid packing (at 4.3 Å) in permanently waved hair samples is indicative of a reduced number of coiled-coil proteins. Fig. 1 ▸(*a*) identified one signal for distances of ∼4.6 Å due to the ring-like scattering at ∼1.4 Å^−1^ in the equatorial direction, which can be related to distances between β sheets (Yu *et al.*, 2017 ▸).

**Figure 3:**
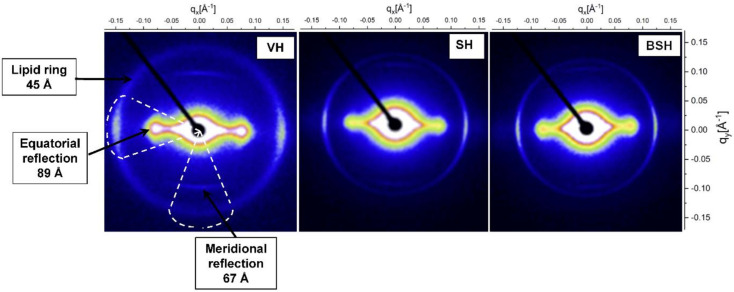
2D images obtained from SAXS zone planes with a sample–detector distance of 98.2 cm for VH, BH (data already published in 2020), SH and BSH.

Fig. 3 ▸ shows the 2D images obtained from the small-angle scattering region (*q* range 0.0125–0.17 Å^−1^), where one can acquire information on the supramolecular arrangements and the interfaces between the structures.

The diffractograms exhibited differences in the anisotropic lipid ring, in the intensity of the signal at 89 Å (equatorial reflection) and in the distribution of the diffuse scattering surrounding the beamstop in both hair tresses treated with straightener. Among these changes, the BH tresses (previously published data; Lima *et al.*, 2020 ▸) showed that oxidative treatment changed only the intensity of the equatorial reflection located at 89 Å. Wade *et al.* (2013 ▸) and Cornwell *et al.* (1994 ▸) related the diffuse scattering to the presence of amorphous/disordered material of the sample and the interfaces between the supramolecular structures. Alterations were also observed in the meridional reflection, shown by shortening in the signal at 67 Å (Fig. 3 ▸; SH and BSH hair samples), which indicates changes in the axial stagger between mol­ecules along the IFs.

**Figure 4:**
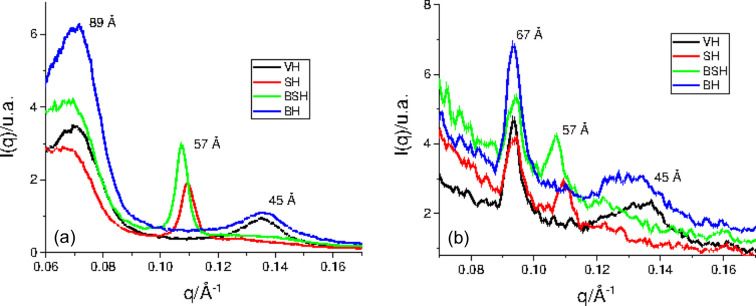
Integrations of the 2D scattering data in the SAXS zone planes (sample–detector distance = 98.2 cm, Cu source): (*a*) equatorial and (*b*) meridional reflections.

Figs. 4 ▸(*a*) and 4 ▸(*b*) show intensity profiles extracted from the SAXS patterns for all the hair samples. The equatorial SAXS zone [Fig. 4 ▸(*a*)] is related to the radial geometry of the filaments and to their lateral packing organization in the matrix.

The strong signal at 89 Å is attributed to the microfibrils’ crystalline lateral organization (Briki *et al.*, 1998 ▸; Fraser *et al.*, 1964 ▸), and this reflection has its shape changed and displaced to smaller *q* values in the straightened hair tresses (SH and BSH groups), differently from what was observed in BH (previously published data; Lima *et al.*, 2020 ▸). These results suggest that the acid treatment changed the IFs and therefore changes the distance between the microfibrils. This fact could be a result of protein denaturation using a hot flat iron (180°C). Indeed, Istrate *et al.* (2009 ▸) demonstrated that the increase of temperature promotes the unfolding of helical domains (IFs), involving the transition from a relatively ordered structure to a more flexible, disorganized, open polypeptide chain. The covalent S–S bonds are very important in this process because they control the strength of the interface (IF matrix) and their breakage may lead to changes in denaturation enthalpy (Δ*H*_D_). These interactions are related to the stability of the IF structure and the unfolding transition of the helical material. A study by Baias *et al.* (2009 ▸) supports the hypothesis that the thermal denaturation pathway occurs simultaneously with the collapse of the scaffold domains, where the α-helix regions go from a relatively compact ordered structure to more flexible, disorganized, open polypeptide chains.

These findings are in the same direction as the DSC data, an interesting tool to investigate the melting of hard α-helix fiber crystallites (Wortmann *et al.*, 2002 ▸). DSC curves and a plot for the enthalpy changes are shown in Figs. S7 and S8. Table 2 ▸ shows the denaturation enthalpy values (Δ*H*_D_) acquired in triplicate.

The samples of straightened hair (SH and BSH groups) had their Δ*H*_D_ value decreased by about 70.0 and 88.0%, respectively, when compared with VH tresses. Previously published data showed that a single application of oxidative bleach [20%(*v*/*v*); 30 min] can cause a minor decrease in the content of liquid crystalline material hair (Lima *et al.*, 2020 ▸). This important decrease in the Δ*H*_D_ values is related to the loss of crystalline material (α-keratin IFs), also verified by WAXS/SAXS, promoting the weakening of the fibers and changes in their mechanical properties, like resistance and elasticity (Wortmann *et al.*, 2002 ▸; Grosvenor *et al.*, 2018 ▸; Monteiro *et al.*, 2005 ▸). Goshiyama *et al.* (2020 ▸) reported a decrease in the tensile strength of ∼55% in hair tresses treated with acid-straightener formulation containing *Glyoxyloyl Carbocysteine (and) Glyoxyloyl Keratin Amino­acids (and) Water* (at pH 1.0 and thermal treatment). These hair samples also had a decrease in tryptophan content and oxidation of the melanin present in the cortex. Boga *et al.* (2014 ▸) reported structure alterations promoted by acid straightening containing glyoxylic acid (pH ≃ 2, thermal treatment) in yak hair keratin. According to the authors, the treatment showed rearrangements in the secondary structure distribution present in the cortex, with decreasing of the α-helix and increasing β-sheet content. The lower Δ*H*_D_ values shown in Table 2 ▸ for the treated hair are therefore associated with the decrease of the α-helix crystallites as mentioned above.

#### 3.1.1. Changes in the lipid arrangements observed in the SAXS patterns

Different types of lipids, present in the hair structure, form organized liquid crystalline structures and can be investigated by SAXS experiments (Bertrand *et al.*, 2003 ▸; Coderch *et al.*, 2007 ▸; Wade *et al.*, 2013 ▸). These regions are usually called CMCs. It is possible to find lipids with a more nonpolar character, such as free fatty acids and cholesterol esters, and also polar ones (Coderch *et al.*, 2007 ▸; Wertz *et al.*, 1986 ▸) as phytosphingosine, fatty acid, ceramide, cholesterol and cholesterol sulfate (Lee *et al.*, 2005 ▸). Some studies indicate that the organized packagings of these lipids give rise to periodicity of 45 Å, shown by a ring-shaped reflection (Yang *et al.*, 2014 ▸; Coderch *et al.*, 2007 ▸) in the SAXS images (Fig. 4 ▸). For lipid bilayers the peak width depends on membrane elasticity and interactions, among a number of other factors (Nagle *et al.*, 1996 ▸). Since the hair CMC is composed of different types of lipid, proteins *etc.*, it is not simple to derive the dependency of the peak width and other structural factors. Therefore, in our analysis, the peak-width estimation was used for the following discussions. From the FWHM value of the 45 Å ring (Scherrer equation; Guinier, 1994 ▸), it can be deduced that the lipids are stacked within ∼500–1000 Å-thick granules. These domains are spread throughout the cortex without any specific orientation versus hair axis (Busson *et al.*, 1999 ▸). Bertrand *et al.* (2003 ▸) reported the existence of a notable difference between the SAXS patterns of cortical and cuticular lipids, demonstrating that it is unlikely that lipids (if present) on the surface of the hair fiber will contribute significantly to the diffraction signal.

Figs. 4 ▸(*a*) and 4 ▸(*b*) show an important change of the lipid liquid crystalline structures in the straightened hair tresses (SH and BSH groups), which is demonstrated by the shifting of their peaks to smaller *q* values in relation to VH, indicating changes in lipid packing. The lipid-ring signal shifted from *d* spacing = 45.5 Å (VH) to *d* spacing = 56.2 and 57.4 Å, for straightened hair samples SH and BSH, respectively. Interestingly, one can clearly see a higher increase in the intensity of the reflection of the planes in the equatorial [Fig. 4 ▸(*a*)] in relation to the meridional [Fig. 4 ▸(*b*)] directions, indicating tendency of the crystalline planes to be oriented preferentially parallel to the hair-fiber axis (Bertrand *et al.*, 2003 ▸).

**Figure 5:**
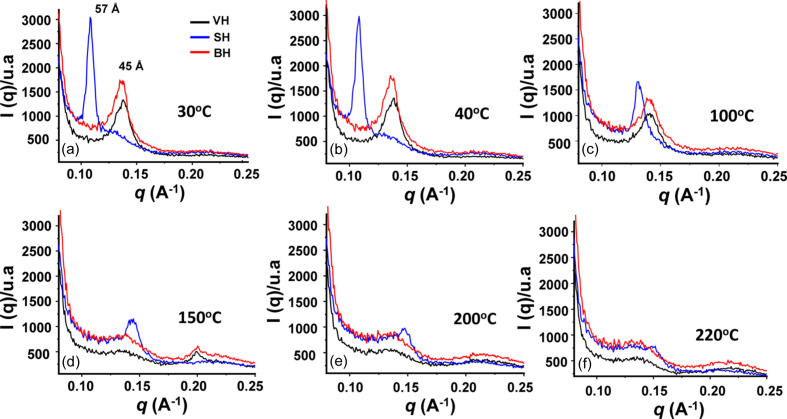
Equatorial integrations of the 2D scattering data in the SAXS zone planes (sample–detector distance = 64 cm) in tresses of hair: VH (in black), BH (in red) and SH (in blue). Measurements were obtained for 1 h at controlled temperatures: (*a*) 30.0 ± 0.2°C, (*b*) 40.0 ± 0.2°C, (*c*) 100 ± 1°C, (*d*) 150 ± 1°C, (*e*) 200 ± 3°C and (*f*) 220 ± 3°C.

To investigate the thermal stability of the liquid crystalline lipid structures inside of the cortex, hair tresses (VH, BH and SH groups) were measured in SAXS experiments under controlled heating from 30 to 220°C. Figs. 5 ▸(*a*)–5 ▸(*f*) show the diffractograms of the SH tresses, where one can see the shift of the peak related to the distance between the lipid layers (anisotropic ring) to lower *q* values as the temperature increases. This behavior is much less pronounced in the BH tresses, which also show a change in reflection intensity at 45 Å. These changes are attributed in the literature (Coderch *et al.*, 2007 ▸; Bertrand *et al.*, 2003 ▸; Cornwell *et al.*, 1994 ▸) to an increase in bilayer distances in the multi-lamellar lipid structures, which suggests swelling of the hair CMC.

These results involving straightening based on the blend of carbonyl-based compounds have not yet been reported in the literature. The findings in this work indicate a change in the lipid organization of the hair fiber with a higher retention of the water inside the hair fiber. This indicates that a large amount of water can be retained in the lipid hair membrane, affecting the lamellar distances. A detailed identification of the peak positions and corresponding periodicities is shown in Table S1 and Fig. S9 of the supporting information.

In other words, with increasing temperature, the reflection at 56 Å shifted to higher *q* values, decreasing the distance between the lipid layers until it practically overlaps the value of the lipid signal in VH, at ∼45 Å, around 220°C. According to Coderch *et al.* (2007 ▸), in contrast to the structural behavior of lipids in other keratinized tissues, hair lipid lamellae have the ability to retain different amounts of water as a function of lipid concentration. Murthy *et al.* (2019 ▸) investigated hydration of the hair fiber using neutron scattering measurements. The authors reported that the lipid spacing increased with hydration of the hair fiber, and it also appears that water is more present outside the microfibrils, in the voids and at the cortex interfaces. The authors also reported that the heat treatments cause a reduction in the S–S bonds, promoting openings in the matrix, which affects the structure and water permeability.

#### 3.1.2. TG–MS measurements

**Figure 6:**
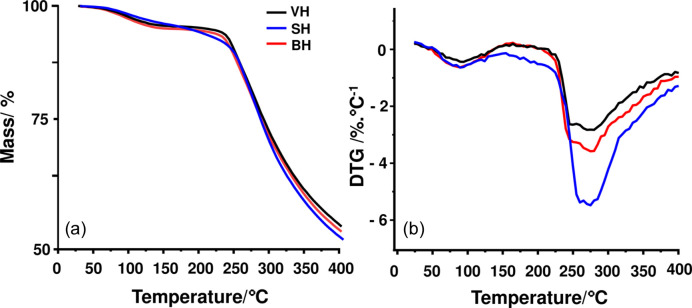
(*a*) TG and (*b*) DTG curves obtained in an open Al_2_O_3_ crucible at 10°C min^−1^ under dynamic air atmosphere (flow rate of 50 ml min^–1^) of samples of VH (in black), BH (in red) and SH (in blue).

We used TG–MS to evaluate if the thermal behavior associated with change in the liquid crystalline lipid granules is associated with mass loss. It was possible to identify the gasses released (obtained by MS) with the mass changes during the increase of the temperature (obtained from TG). Figs. 6 ▸(*a*) and 6 ▸(*b*) show the overlap of TG and the first derivative of the TG curve with respect to time (DTG) (respectively) of samples of VH, BH and SH tresses.

The DTG curves [Fig. 6 ▸(*b*)] show that the dehydration profile is different (first mass-loss event) between the samples, and the water content (data in Table 3 ▸) is eliminated more slowly in the SH sample. In the second event, its mass loss (keratin decomposition) is different from that shown in VH and BH.

**Figure 7:**
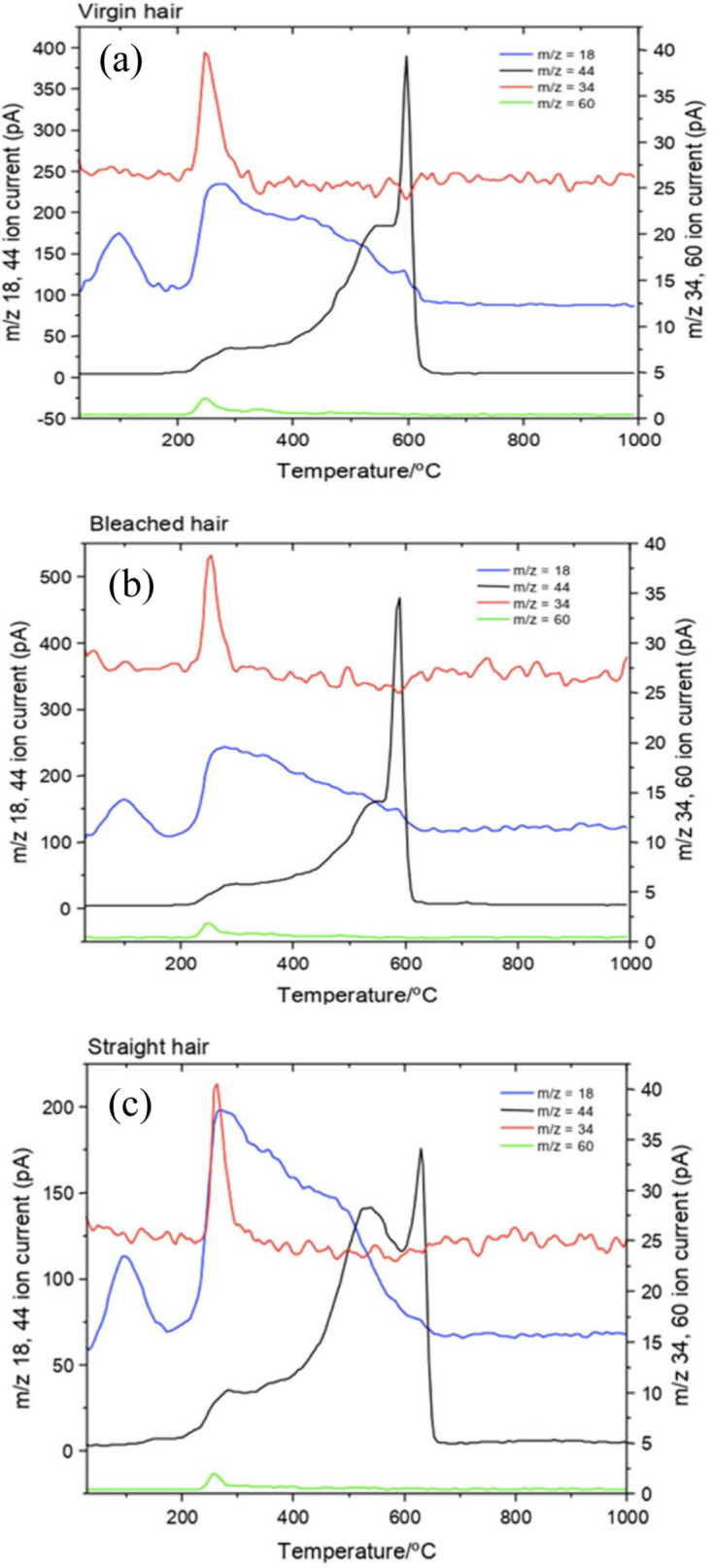
Evolution of gasses during the TG analysis of samples of hair: (*a*) VH, (*b*) BH and (*c*) SH. The gasses involved were H_2_O (*m*/*z* = 18), CO_2_ (*m*/*z* = 44), SCO (*m*/*z* = 60) and H_2_S (*m*/*z* = 34).

Brebu & Spiridon (2011 ▸) have shown that the variation of *m*/*z* mass numbers (mass/charge ratio) with temperature during TG analysis offers valuable information on the volatile compounds formed during thermal degradation of hair samples. In the present work, the MS spectra [Figs. 7 ▸(*a*), 7 ▸(*b*) and 7 ▸(*c*)] showed that, in the range of 25 to ∼125°C, only water (*m*/*z* = 18) was released in the SH sample, which confirms that the decrease in distance between the lipid layers observed in the SAXS *in situ* measurements (Fig. 5 ▸) was caused by the loss of water in the hair tresses during heating.

This behavior of the CMC shows that this microstructure has a fundamental role in the retention of water inside the hair shaft. Other gasses were also observed in the mass spectra of these hair samples [Figs. 7 ▸(*a*), 7 ▸(*b*) and 7 ▸(*c*)]: CO_2_ (*m*/*z* = 44), SCO (*m*/*z* = 60) and H_2_S (*m*/*z* = 34). Lima *et al.* (2019 ▸) also detected the formation of classes of heteroatom-containing compounds (sulfides and thiols) during the heating of hair, originating from the thermal decomposition of amino acids.

However, in our research, the MS spectra of the SH sample showed a release of CO_2_ (*m*/*z* = 44) at ∼177°C, about 26°C before the VH sample (Table 3 ▸). This behavior is seen in the TG/DTG curves [in detail in Fig. 6 ▸(*b*)] of the SH sample, which show the beginning of thermal decomposition – second mass-loss event – before the VH sample.

To explain the higher ‘confinement’ of the water molecules between the lipid bilayers observed for SH, we can consider the following hypotheses:

(i) the acid straightening associated with the heat from the thermal device forms a film around the fiber [according to Goshiyama *et al.* (2020 ▸)], hampering the ‘exit’ of the water, which is verified by TG data;

(ii) the deposition of the acid active (or its by-products) on the surface and/or in the interior of the fiber promotes its rigidity [according to Colenci (2017 ▸) and Goshiyama *et al.* (2020 ▸)], and the changes in the protein structure inside the fiber consequently cause a change in the ordination (packing) of the fiber’s lipid composition, according to data obtained by WAXS/SAXS measurements.

### 3.2. Neutron scattering

**Figure 8:**
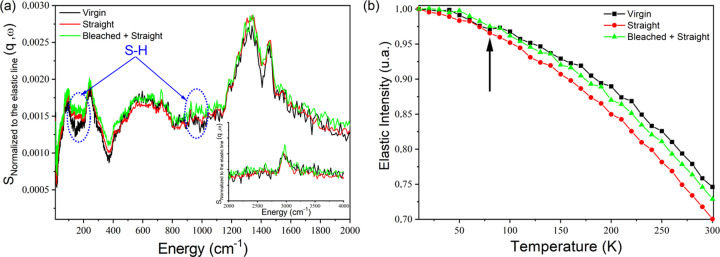
(*a*) Normalized evolution of immobile protons on the nanosecond timescale obtained from data collected using IRIS at ISIS. The error bars are within the size of the symbols. (*b*) Normalized vibrational spectra obtained at 10 K using the vibrational spectrometer TOSCA. VH samples are shown in black, SH samples in red and BSH samples in green. In (*a*) the intensity of the signal is directly proportional to the phonon density. In (*b*) the anomalous decrease of the elastic intensity, indicated by an arrow and occurring around 80 K, is indicative of the onset of some type of diffusive motion that is faster than the instrumental time resolution (40 ps). Blue dotted circles indicate vibrational modes attributed to S–H torsion (∼150 cm^−1^) and S–H bending (∼950 cm^−1^) in l-cysteine (Bordallo *et al.*, 2010 ▸; Pawlukojc *et al.*, 2005 ▸).

QENS and INS data from hair samples were used in this study to investigate the hydrogen mobility arising from proteins, lipids and water in the chemically changed structure. Figs. 8 ▸(*a*) and 8 ▸(*b*) show the results. Collagen is the main protein constituent of a wide variety of connective tissues in animals. For the INS data, the water bound to collagen was assigned to bands with intensity at ∼50 cm^−1^ and a broad band between 500 and 800 cm^−1^ (Parker, 2001 ▸). The first vibration is assigned to the interhelical water hydrated sample and the second signal to modes from both the tightly bound and interhelical water. Fig. 8 ▸(*a*) shows an alteration in the intensity of the vibrational modes assigned to the hydrogen atoms around 500–800 cm^−1^ of the INS spectra of the hair tresses. In this region, a slight difference in the intensity between the types of hair was observed, while differences at ∼50 cm^−1^ were not detected. This is an indication that protein denaturation in the SH and BSH samples did not affect the water in the microfibrils. Kamath *et al.* (2014 ▸) demonstrated that the amount of water adsorbed through hydrogen bonding and exchanged with amine and amide nitro­gen in the spaces between the IFs (interhelical) appears to be relatively small compared with that in mesoporous regions as multimolecular layers. If one correlates these results with the SAXS data for the straightened hair tresses (SH and BSH), it is possible to conclude that the water molecules appear to be, preferably, between the lipidic bilayers (CMC), located in the outer region of the microfibrils (hair matrix).

Even though it was not possible to observe the peak of amide I at 1650 cm^−1^, since this is expected to be weak in INS (Middendorf *et al.*, 1995 ▸), other important features were detected in the spectra. For example, there are alterations in the intensity at ∼1330 and 1450 cm^−1^ for the treated hair tresses, which were assigned by Festa *et al.* (2019 ▸) to lipid components and by Parker (2001 ▸) to displacement of the groups CH_2_ wag, CH_2_ twist, CH_2_ bend, CH bend, and CH_3_ symmetric and antisymmetric bend. This observation is also reflected by the changes in EFW results [Fig. 8 ▸(*a*)], represented as mean square displacements (Bordallo *et al.*, 2010 ▸), observed for the averaged *q* values. The data show the increase in proton mobility with the different treatments, observed by the greater and continuous decay of the curves corresponding to each sample. This means that the structure of VH is more rigid, where hydrogen atoms (from water, protein, lipids) are less mobile.

Comparing the hair treated tresses, the QENS curve related to BSH remained between the BH QENS curve and the SH QENS curve. This shows that the overlapping treatments promote a rearrangement in the hydrogenated molecules. Taddei *et al.* (2015 ▸) detected changes in the average tyrosine environment and its hydrogen-bonding state with increasing glyoxylic acid incorporation, making the tyrosine residues more exposed. These findings may explain the greater proton mobility obtained in this research. In addition, the serine and lysine amino acidic residues appeared to be involved in the reaction with glyoxylic acid resulting in the formation of iminic species. In this sense, the pH of the formulation appears an important factor for subsequent reactions of the hair treatments, even after washing.

The above results indicate that the ‘starting point’ of the BH tresses (which were exposed to pH ≃ 10) and SH tresses (at pH ≃ 1) promotes important structural changes when compared with VH (pH ≃ 5). These explanations are also supported by the HRXRM and USAXS data, which show a significant difference in the quantified porosity for the sample of BSH, as shown in the next section.

#### 3.2.1. High-resolution 3D X-ray microscopy and computed tomography (HRXRM) and USAXS

We used the ZEISS Xradia Versa XRM-510 microscope to observe and quantify the porosities of hair fibers of groups VH, BH and SH. Table 4 ▸ shows these values expressed in percentages. The size distributions were also obtained and are shown in Figs. S3–S6. The obtained values are within the resolution limit of the technique and therefore one can compare the overall results among the samples in the batch but not the exact values themselves. From the percentage values, BSH showed the highest values among the samples. In fact, these increases in porosity corroborate findings in the literature by other analytical techniques, which show that bleaching (oxidative damage) makes the hair fiber more susceptible to damage from subsequent treatments. This is due to, in addition to melanin degradation (Robbins, 2012 ▸; Wolfram *et al.*, 1970 ▸), bleaching caused by hydrogen peroxide (which occurs at a pH close to 10), causing detachment of cuticular layers and removing the lipid 18-MEA (Masukawa *et al.*, 2005 ▸), promoting a greater capacity for absorption of cosmetic actives, and therefore accelerating the kinetics of the action of subsequent treatments (Martins Junior, 2015 ▸). In addition, bleaching leads to alteration in the water retention of the hair (Lima *et al.*, 2020 ▸) and an increase in cysteic acid residues in the fiber, causing a significant change in the distribution of cross-electrostatic bonds (Wolfram *et al.*, 1970 ▸). Wolfram *et al.* (1970 ▸) suggest that the swelling of BH is directly related to the overall chemical modification of keratin by H_2_O_2_ and may represent a convenient means for the assessment of the extent of damage.

**Figure 9:**
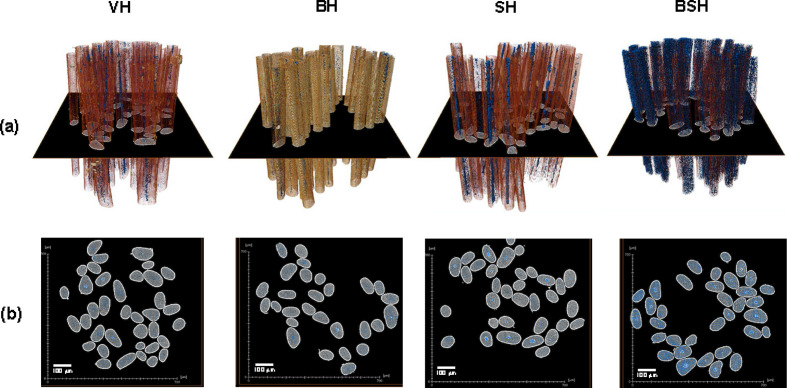
(*a*) 3D images showing the cuts used to obtain the porosity values of the hair fibers and (*b*) 2D images of the same cuts.

Figs. 9 ▸(*a*) and 9 ▸(*b*) show 3D and 2D images (respectively) obtained by X-ray microtomography measurements of the hair fibers. The images in the 2D part represent a section (in the black axial plane) of the fiber arrangements shown in Fig. 9 ▸(*a*).

The porosity is represented by the blue dots. It is possible to observe a significant difference in the increase in porosity between the fibers subjected to bleaching followed by straightening (BSH) and VH.

**Figure 10:**
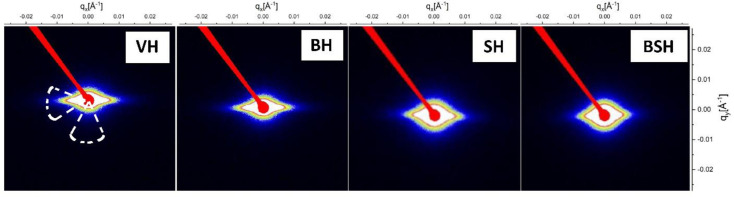
2D images obtained from USAXS zone planes for the sample hair types. Sample–detector distance = 650 cm.

The USAXS data for each hair sample are shown in Fig. 10 ▸. The 2D images show an anisotropic scattering, which indicates the presence of anisotropic features in the hair fibers for the USAXS region.

**Figure 11:**
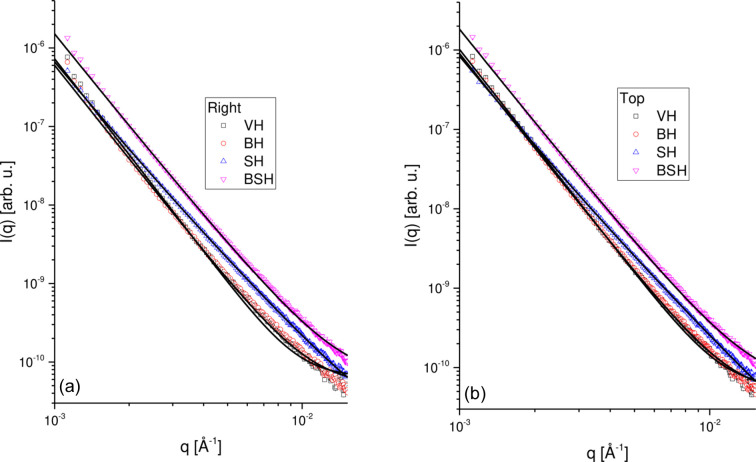
1D (*a*) vertical and (*b*) horizontal cuts of USAXS data. Symbols: experimental data. Solid lines: theoretical curves.

To attain a quantitative analysis of the scattering, vertical and horizontal cuts were performed on the USAXS data. The 1D curves are shown in Fig. 11 ▸. A model for fractal structures composed of spherical subunits was used to describe the data. In this model the scattering intensity is given by

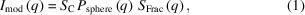

where *S*_C_ is an overall scale factor and *P*_sphere_(*q*) is the intensity form factor of spherical particles with radius *R*:

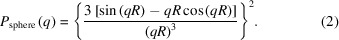

The structure factor of fractal aggregates with fractal dimension *D*, overall fractal size ξ and subunit characteristic radius *R* is given by (Teixeira, 1988 ▸)

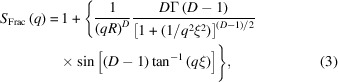

where Γ(*x*) is the Gamma function. Another possibility is having surface fractal structures (Anitas, 2020 ▸; Bale & Schmidt, 1984 ▸). In this model, pores are large (not visible on the experimental *q* range), and therefore the contribution comes mainly from the inner surface. In this case, the scattering intensity is given by

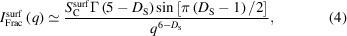

where 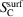 is an overall scale factor and *D*_S_ is the fractal dimension for the surface. Therefore, the main point of the fractal analysis is the fact that for volume fractals one has 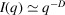 and for surface fractals one has 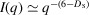 . As shown in Fig. 9 ▸, the hair fibers have pores, which give the main contribution to the USAXS data. Due to resolution limits on the HRXRM technique, it is not possible to see pores smaller than ∼3 µm. USAXS, on the other hand, is certainly sensible to pores smaller than this value. Therefore, the fractal contributions detected by USAXS might come from volume fractals for the smaller pores within the matrix and also from the inner surface from the large pores. It is beyond the scope of this article to give a deeper description of the pores, but the results obtained from the data analysis give structural insights on the pore distribution in the system.

The results obtained from the modeling analysis are summarized in Table 5 ▸. Since the curves are related to USAXS data, the optimization of the small subunit sizes (used in either the form factor or the fractal structure factor) was very unstable. Therefore, a typical value of 15 Å was used, which provided reasonable fits. For either the vertical or the horizontal cuts, we obtained slopes of ∼3.0, which could indicate the formation of volume fractal structures (*D* = 3.0) or rough surface fractals (*D*_S_ = 6 − *D* = 3.0). For the volume fractal hypothesis, the obtained fractal sizes indicate an interesting behavior for each hair sample. For VH we obtained larger fractal sizes for the horizontal cuts (perpendicular to the hair-fiber alignment). Similar results were observed for BH but with larger sizes, indicating the increase of the overall pore sizes. For SH, on the other hand, the obtained sizes for the vertical or horizontal cuts provide similar sizes for the fractal sizes, with values slightly larger than those obtained for VH.

Finally, for BSH, the results indicate the formation of larger fractal aggregates in the vertical direction than in the horizontal direction. The analysis of the overall scale factor *S*_C_ is also important since it is related to the overall pore fraction in the system. We obtained the largest values for the BSH sample, indicating that the fraction of the pores is larger in this sample than in the other cases. The pore-size distributions (Figs. S3–S6) also indicate an increase of large pore fractions in the BSH samples. All in all, the USAXS and HRXRM data show a nice correlation and indicate the presence of pores inside of the hair fibers, which varies according to the type of treatment.

## 4. Conclusions

In this work, we presented a multidisciplinary investigation combining structural, thermodynamic, vibrational, microscopic and thermogravimetric data for the investigation of human hair tresses. It was shown that the hair straightener containing a blend of carbonyl-based compounds modified microstructural components of the fiber. These data corroborated those reported in the literature, evidencing that this type of procedure causes marked changes in the hair fiber cortex. The main changes include (i) denaturation of the IFs (keratin α-helix); (ii) swelling of the CMC, shown by the distancing of the lipid bilayers; (iii) greater susceptibility to the cortical porosity of the fiber when applying this type of straightener to previously bleached hair; (iv) alteration in the groups (CH_2_) of the lipids; and (v) increased proton mobility in the structure.

The presence of pores inside the hair fibers and their variation according to the type of treatment on the fiber were investigated using HRXRM and USAXS. The results indicated the variation of the pore sizes and fraction depending on the treatment of the fiber. The pore fraction is directly related to the fiber mechanical resistance and our investigations provide important results on this point. In addition, it was possible to verify that the lipid bilayers of the cortex have a significant importance in water retention inside the fiber.

All these data associated with those reported in the literature demonstrated that the action mechanism of acid straightener products on hair is complex. The approach proposed in this work is applicable for a detailed evaluation of alterations to microstructure at different length scales inside the hair fiber prompted by different cosmetic treatments. Interestingly, these techniques involve non-destructive testing of samples. The results shown in this article can provide support to companies, researchers and professionals in hair cosmetology for proper understanding of the changes caused by acid actives in hair fibers under heating.

## Supplementary Material

Supporting information. DOI: 10.1107/S1600576723005599/tj5033sup1.pdf

## Figures and Tables

**Table 1:** Sample–detector distance values (*D*_SD_) used and their respective ranges for the scattering-vector values (*q*)

		Vector modulus *q* (Å^−1^)	
*D*_SD_ (cm)	Source	*q*_min_	*q*_max_
650 (USAXS)	Cr	0.001	0.030
98.2 (SAXS)	Cu	0.0125	0.170
14.3 (WAXS)	Cu	0.085	1.500
14.3 (WAXS)	Mo	0.160	3.000

**Table 2:** Enthalpy (Δ*H*_D_) values of the hair samples obtained from the DSC data Δ*H*_D_ was obtained by integrating the area under the denaturation peak.

Sample	Δ*H*_D_ ± standard deviation (J g^−1^)
VH	17.5 ± 0.9
BH†	15.0 ± 1.0
SH	5.2 ± 0.6
BSH	2.3 ± 0.6

† Data already published in 2020.

**Table 3:** TG and MS results: range temperature (Δ*T*), mass loss (Δ*m*) and released gasses of each event of the hair sample

Sample hair	Technique	Event		Δ*T* (°C)†	Δ*m* (%)†
VH	TG	Dehydration		30–165	4.7
		Decomposition		203–638	92.4
	MS	Gas	CO_2_	From 203	–
			SCO	214–275	–
			H_2_S	222–319	–

BH	TG	Dehydration		30–181	5.1
		Decomposition		211–623	91.5
	MS	Gas	CO_2_	From 211	–
			SCO	214–304	–
			H_2_S	217–297	–

SH	TG	Dehydration		30–173	5.1
		Decomposition		177–668	92.9
	MS	Gas	CO_2_	From 177	–
			SCO	221–296	–
			H_2_S	232–319	–

† Average of duplicate measurements.

**Table 4:** Porosity data (maximum and minimum values)

		% Pore volume		
Sample	Fiber number	Minimum	Maximum	Average + standard deviation
VH	37	1.65	5.53	3.26 ± 1.05
BH	28	1.53	7.93	3.43 ± 1.50
SH	34	0.70	8.27	3.51 ± 1.83
BSH	34	6.96	20.89	11.97 ± 3.62

**Table 5:** Structural parameters obtained from the modeling of USAXS data The fractal subunit radius and the subunit radius for the form factor were set to 15 Å.

Cut direction	Sample	*S*_C_ (10^−10^)	*D* †	ξ (10^3^ Å)
Horizontal	VH	1.3 (8)	3.000 (1)	297 (100)
	BH	2 (1)	3.000 (1)	556 (100)
	SH	1.1 (4)	3.000 (1)	372 (100)
	BSH	3 (1)	3.000 (1)	357 (100)

Vertical	VH	1.0 (6)	3.000 (1)	167 (70)
	BH	1.3 (8)	3.000 (1)	265 (100)
	SH	1.2 (3)	3.000 (1)	321 (100)
	BSH	2 (1)	3.000 (1)	185 (80)

† The fractal dimension *D*_S_ = 6 − *D* also has a value close to 3.0.
