# Supplementary material for: Alterations promoted by acid straightening and/or bleaching in hair microstructures
Source: J Appl Crystallogr. 2023 Aug 1;56(Pt 4):1002–14. doi: 10.1107/S1600576723005599 (PMC10405601; doi:10.1107/S1600576723005599)
Supplement: Supplementary file 1 [file j-56-01002-sup1.pdf]

JOURNAL OF APPLIED CRYSTALLOGRAPHY

Volume 56 (2023)

Supporting information for article:

Alterations promoted by acid straightening and/or bleaching in hair microstructures

C. R. R. C. Lima, R. J. S. Lima, A. C. C. Bandeira, R. A. A. Couto, M. V. R. Velasco, H. N. Bordallo and C. L. P. Oliveira

Alterations promoted by acid straightening and/or bleaching in hair microstructures

C. R. R. C. Lima[1], R. J. S. Lima[2,3], A.C. Bandeira[1], R. A. A. Couto[4], M. V. R. Velasco[5], H. N. Bordallo[3,6*], C. L. P. Oliveira[1*]

[1] Institute of Physics, University of São Paulo, São Paulo 05508-090, SP, Brazil

[2] Academic Unit of Physics, Federal University of Campina Grande, Campina Grande 58429-900, PB, Brazil

[3] Niels Bohr Institute, University of Copenhagen, 2300 Copenhagen, Denmark

[4] Institute of Chemistry, University of São Paulo, São Paulo, Brazil

[5] Faculty of Pharmaceutical Sciences, University of São Paulo, São Paulo 05508-000, SP, Brazil

[6] European Spallation Source ESS ERIC, PO Box 176, SE-221 00 Lund, Sweden

Corresponding authors: crislpo@if.usp.br and bordallo@nbi.ku.dk

Supplementary Information

1- Hair tresses preparation and mounting for scattering measurements

As described in the main text, after being subject to cosmetic treatments, the hair tresses were separated into 4 groups. Of these, approximately 100 hair fibers of each type (each group) were cut into around 5 cm long and placed in the sample holder as shown in the image below. SAXS data were collected in a transmission geometry and the hair fibers were all carefully put all parallel along the axis of the hair. The apparatus used to mount the hair fibers in the experiment is in Figure S*1*.

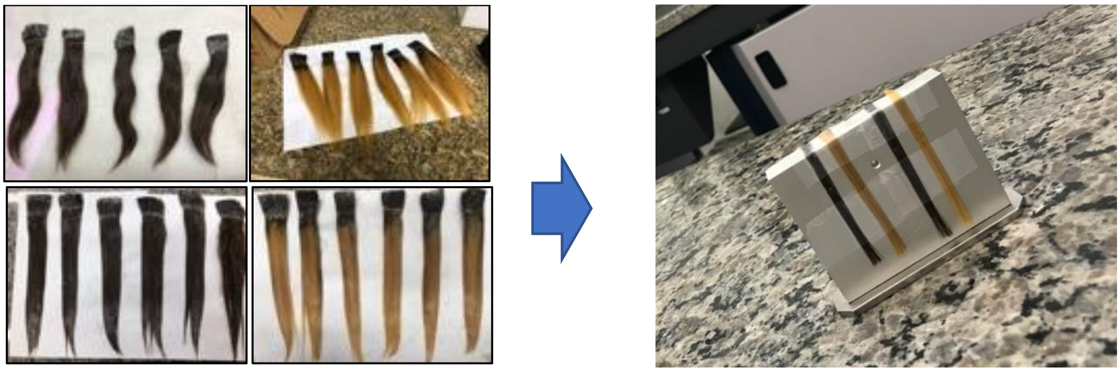

Figure S1 – Hair tresses preparation and mounting for X-Ray scattering measurements

A similar arrangement was used for the measurements on the temperature variation measurement. The hair fibers were again carefully put all parallel along the axis of the hair and mounted on the sample holder as shown in Figure S*2*.

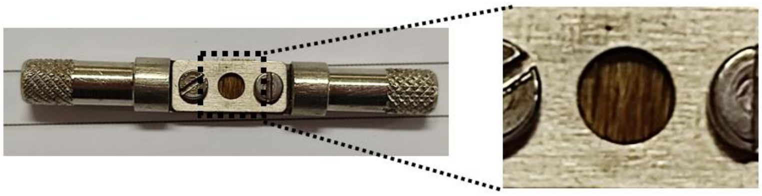

Figure S2 – Hair tresses preparation and mounting for X-Ray scattering measurements with temperature variation.

2- Size distributions from High resolution 3D X-ray microscopy

The size distribution values are within the resolution limit of the technique and therefore one can compare the overall results among the samples in the batch but not the exact values itself. That´s why the indications for the percentage values were taken in the analysis.

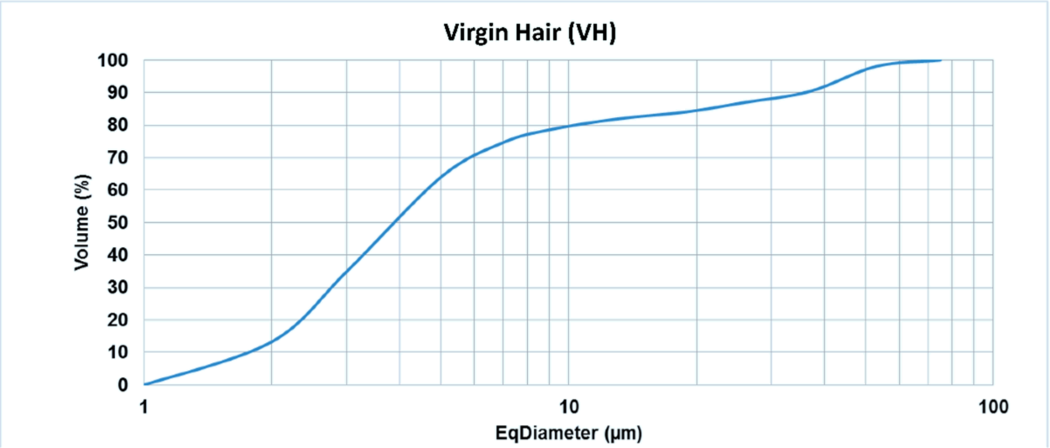

Figure S3 – Size distribution obtained from HRXRM analysis for Virgin Hair (VH).

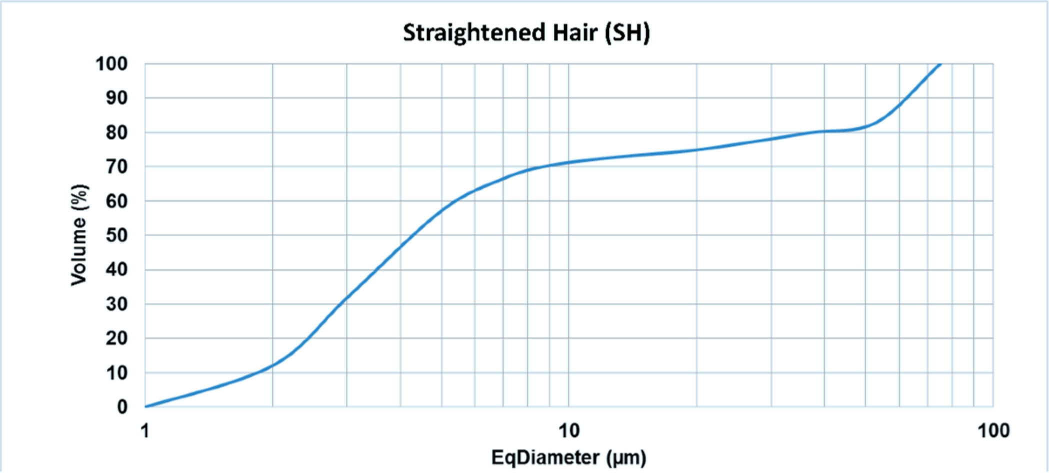

Figure S4 – Size distribution obtained from HRXRM analysis for Straightened Hair (SH).

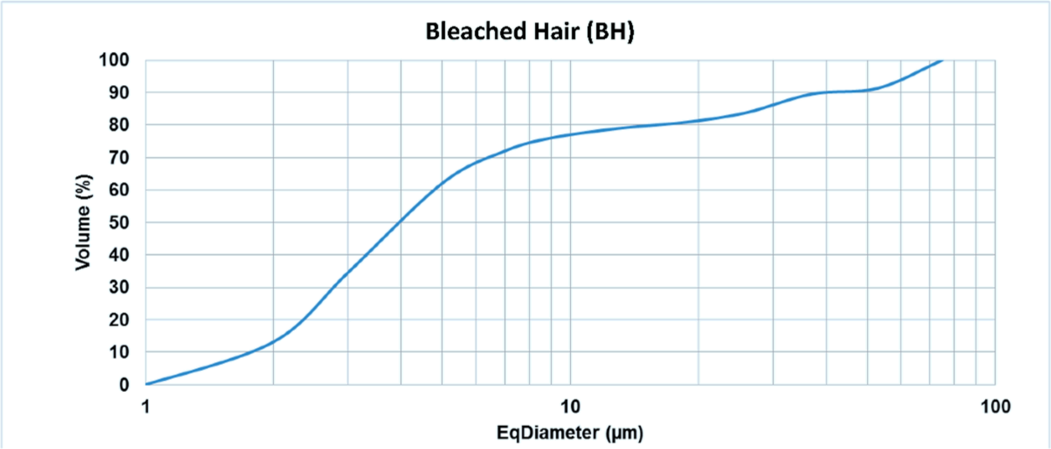

Figure S5 – Size distribution obtained from HRXRM analysis for Bleached Hair (BH).

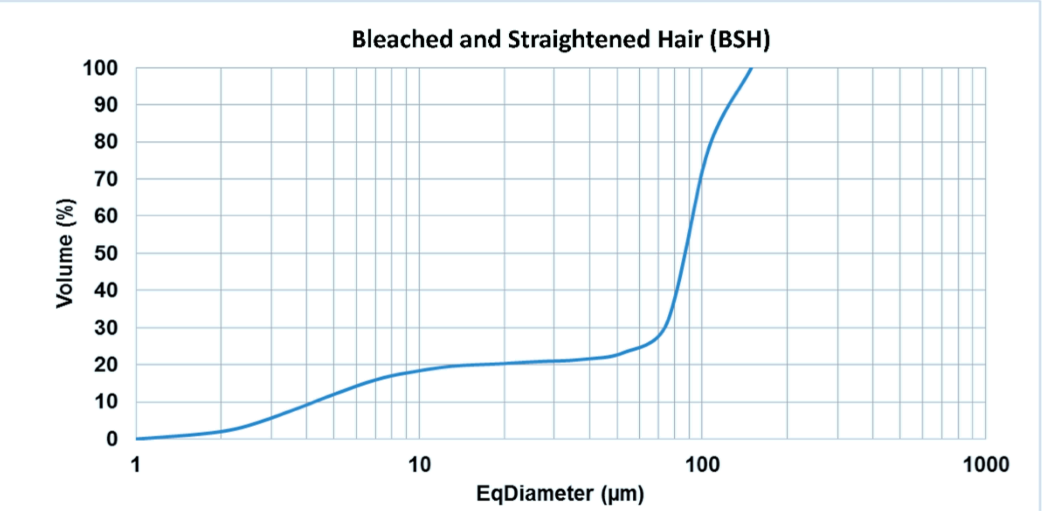

Figure S6 – Size distribution obtained from HRXRM analysis for Bleached and Straightened Hair (BSH).

3- Differential scanning calorimetry data and enthalpy plots

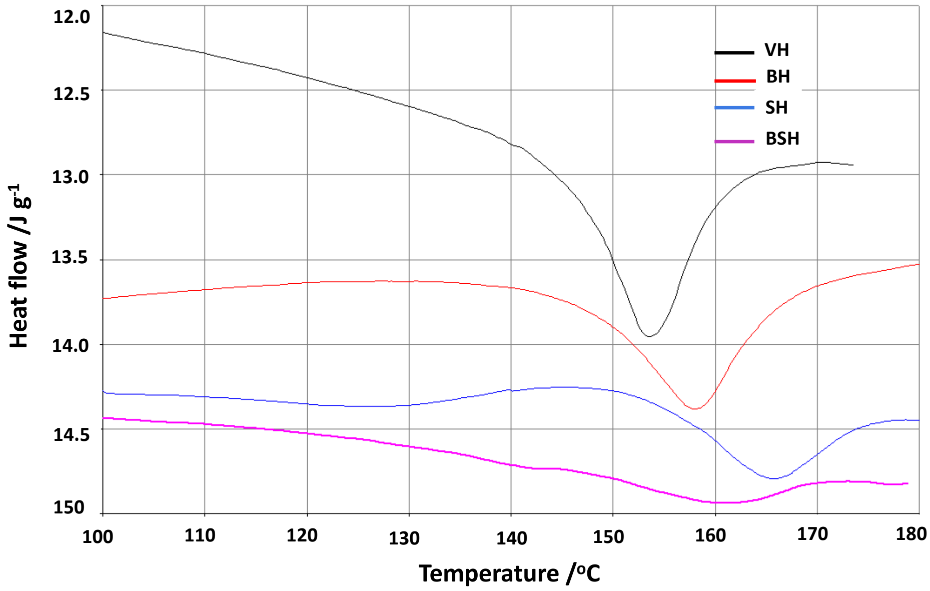

Figure S7 – Overlap of the DSC curves of each sample (one measurement).

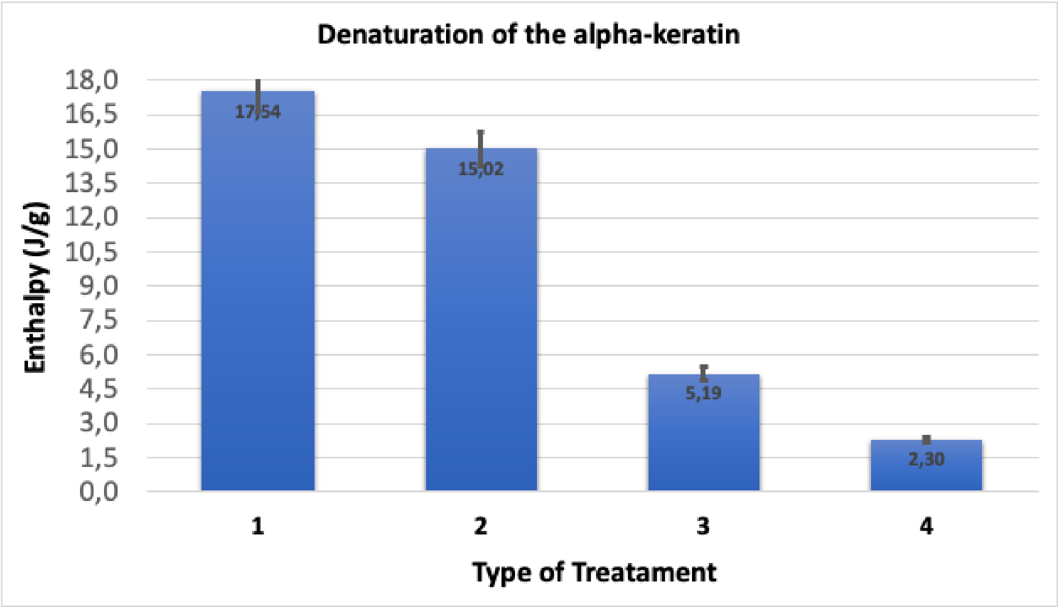

Figure S8 - Enthalpy data (in triplicate). (1) (VH) virgin hair; (2) (BH) bleached hair; (3) (SH) straightened hair and (4) (BSH) bleached and straight hair.

4- Peak analysis for temperature variation on SAXS studies

The peaks indicated in figure 5 were fitted using a Gaussian function and the peak position was determinated. Using the Bragg law, $D_{peak} = \frac{2\pi}{q_{peak}}$ we obtained the values shown in table S1 :

Table S1 – Peak positions and corresponding periodicities calculated

Temperature °C	Parameter	Sample		
		VH	BH	SH
30	q_{peak} (Å^{-1})	0.13746(2)	0.13522(2)	0.10838(5)
	D_{peak} (Å)	45.71(7)	45.47(7)	57.97(4)
40	q_{peak} (Å^{-1})	0.13732(7)	0.1357(2)	0.1080(1)
	D_{peak} (Å)	45.76(5)	46.32(6)	58.18(4)
100	q_{peak} (Å^{-1})	0.1404(1)	0.1401(1)	0.1316(1)
	D_{peak} (Å)	44.75(5)	44.84(5)	47.76(4)
150	q_{peak} (Å^{-1})	0.1334(8)	0.1347(7)	0.1432(1)
	D_{peak} (Å)	47.1(3)	46.7(2)	43.9(1)
200	q_{peak} (Å^{-1})	0.132(2)	0.1337(7)	0.1467(2)
	D_{peak} (Å)	47.6(8)	47.0(2)	42.8(1)
220*	q_{peak} (Å^{-1})	---	---	---
	D_{peak} (Å)	---	---	---

* For this temperature it was not possible to identify the peaks.

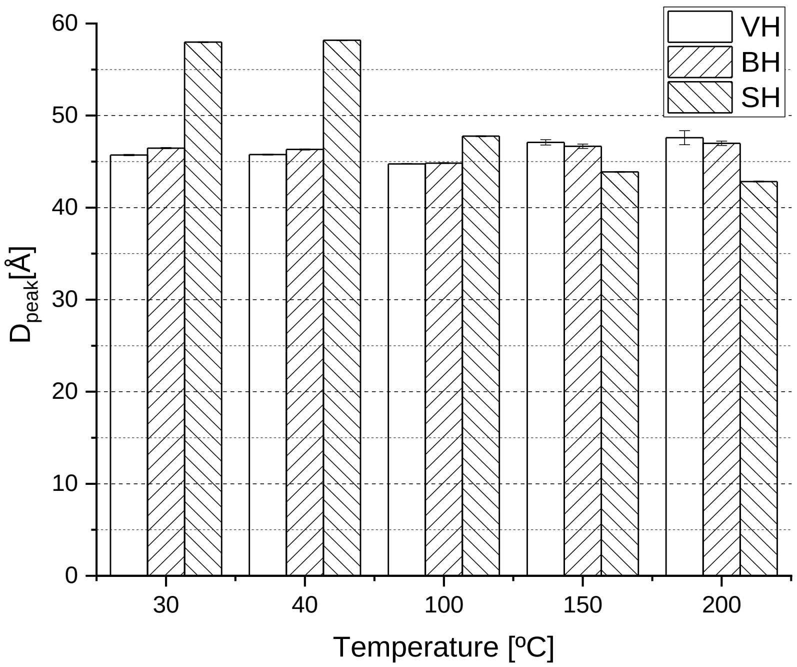

Figure S9 – Lamellar distance calculated for the CMC-related peak.
